# Clinical Phenotype and Prognostic Significance of Frailty in Transthyretin Cardiac Amyloidosis

**DOI:** 10.1016/j.jaccao.2025.01.018

**Published:** 2025-03-12

**Authors:** Carlo Fumagalli, Adam Ioannou, Francesco Cappelli, Mathew S. Maurer, Yousuf Razvi, Aldostefano Porcari, Mattia Zampieri, Federico Perfetto, Muhammad U. Rauf, Ana Martinez-Naharro, Lucia Venneri, Aviva Petrie, Carol Whelan, Ashutosh Wechalekar, Helen Lachmann, Philip N. Hawkins, Iacopo Olivotto, Raffaele Marfella, Andrea Ungar, Niccolò Marchionni, Julian D. Gillmore, Marianna Fontana

**Affiliations:** a National Amyloidosis Centre, University College London, London, United Kingdom; b Tuscan Regional Amyloidosis Centre, Careggi University Hospital, Florence, Italy; c Department of Advanced Medical and Surgical Sciences, University of Campania Luigi Vanvitelli, Naples, Italy; d Cardiac Amyloidosis Program, New York-Presbyterian Hospital, Columbia University Irving Medical Center, New York, New York, USA; e Center for Diagnosis and Treatment of Cardiomyopathies, Cardiovascular Department, Azienda Sanitaria Universitaria Giuliano-Isontina, University of Trieste, Trieste, Italy; f Meyer Children’s Hospital, IRCCS, Florence, Italy; g University College London, London, United Kingdom; h Department of Experimental and Clinical Medicine, University of Florence, Florence, Italy

**Keywords:** amyloidosis, ATTR-CA, cardiomyopathy, care delivery model frailty, outcomes, therapy

## Abstract

**Background:**

The prevalence and clinical impact of frailty in transthyretin cardiac amyloidosis (ATTR-CA) remains poorly characterized.

**Objectives:**

This study aimed to evaluate the prevalence, clinical determinants, and prognostic significance of frailty in a large cohort of patients with ATTR-CA.

**Methods:**

Frailty was assessed in 880 patients with ATTR-CA (median age 80 years [Q1-Q3: 75-84 years], 719 [81.7%] male) using the Clinical Frailty Scale (CFS). Frailty was analyzed as a continuous variable and categorized as CFS 1 to 3, CFS 4 or 5, CFS 6 or 7, and CFS 8 or 9.

**Results:**

Frailty was observed in 502 (57.1%) patients (CFS 4 or 5: 364 [41.4%]; CFS 6 or 7: 129 [14.7%]; CFS 8 or 9: 9 [1.0%]). Independent predictors of worsening frailty included older age, female sex, non-p.(V142I) hereditary ATTR-CA variants, and National Amyloidosis Centre stage 3 disease. Mortality rates increased incrementally with frailty severity (deaths per 100 person-years: 2.9 vs 11.0 vs 21.1 vs 40.9; log-rank *P* < 0.001). Frailty was independently associated with higher mortality risk across all age groups, genotypes, and disease stages.

**Conclusions:**

Frailty is common in ATTR-CA and is independently linked to increased mortality risk. Incorporating frailty assessment alongside traditional markers enhances prognostication across genotypes and disease severities, particularly for short-term risk estimation.

Transthyretin cardiac amyloidosis (ATTR-CA) is a life-threatening progressive disease caused by extracellular deposition of misfolded or cleaved transthyretin (TTR) protein.^1^ The sporadic, noninherited, wild-type form (wild-type transthyretin cardiac amyloidosis [wtATTR-CA]) primarily affects older, predominantly male individuals, whereas the hereditary form (hereditary transthyretin cardiac amyloidosis [hATTR-CA]) presents with a variable clinical phenotype, often involving both cardiomyopathy and polyneuropathy.^1^^,^^2^ Recent advances in cardiac imaging and increased clinical awareness have led to a significant rise in diagnoses, with patients being identified earlier in the disease course. Despite earlier diagnosis, most patients are older and have a greater burden of comorbidities.^1^^,^^3^

Once considered a chronic and fatal disease without specific treatment options, ATTR-CA is now a potentially treatable condition. Whereas some patients benefit from disease-modifying therapies,^4^^,^^5^ others face competing risks—both cardiovascular and noncardiovascular—that influence outcomes beyond amyloid burden. Nonamyloid causes of morbidity and mortality include heart failure (HF) from other etiologies (eg, ischemic heart disease), renal dysfunction, cerebrovascular disease, and cancer. Consequently, a thorough and comprehensive evaluation is often necessary.^6^

Frailty is an increasingly recognized syndrome associated with aging and comorbidities.^7^^,^^8^ It is characterized by a decline in homeostatic reserve across multiple physiological systems, resulting in greater vulnerability to stressors.^9^ In the context of chronic HF, frailty is common. Although HF and frailty are distinct conditions, they share several pathophysiological mechanisms, including the dysregulation of metabolic, inflammatory, and neurohormonal pathways.^10^ Despite these shared mechanisms, frailty independently increases the risk of HF hospitalizations and cardiovascular mortality.^11^, ^12^, ^13^ Patients with ATTR-CA are increasingly diagnosed at older ages and with more comorbidities. However, despite the well-established connection between HF and frailty, the significance of frailty in ATTR-CA remains poorly characterized.

This study aimed to evaluate the prevalence, clinical phenotype, and prognostic significance of frailty in a large cohort of patients with ATTR-CA.

## Methods

This retrospective, multinational, observational study included patients diagnosed with ATTR-CA between September 2019 and March 2023 at 2 specialist referral centers: the National Amyloidosis Centre (United Kingdom) and the Referral Centre for Cardiac Amyloidosis, Careggi University Hospital (Italy). Patients were eligible for inclusion if they underwent a comprehensive frailty assessment at baseline. The diagnosis and risk stratification of ATTR-CA were based on validated diagnostic and prognostic criteria.^14^^,^^15^ All patients underwent genetic sequencing of the *TTR* gene. The study was conducted in accordance with the Declaration of Helsinki.

As a retrospective cohort study, frailty assessments, including the Clinical Frailty Scale (CFS), were performed as part of routine clinical care for older patients, rather than for research purposes. Consequently, additional informed consent was not required. The study received approval from the Institutional Review Boards of the participating hospitals, which granted a waiver for informed consent due to the retrospective nature of the data collection and reliance on standard clinical care practices.

### Frailty assessment

Frailty was assessed by trained personnel (either geriatricians or qualified healthcare providers) using the CFS developed by Rockwood et al^16^ as previously described. The CFS, implemented starting in September 2019, is a widely recognized tool that assesses frailty based on a patient’s ability to perform daily living activities, including both instrumental and basic tasks (Supplemental Table 1). The CFS score ranges from 1 to 9; lower scores indicate physical fitness and higher scores reflect worsening functional performance, decline, and potential disability.

To facilitate interpretation and application in clinical practice, frailty profiles were described as both a continuous nominal variable and an ordinal variable, categorized into clinically meaningful distinctions^17^^,^^18^:

• CFS 1 to 3: no impairment in daily living activities.
• CFS 4 or 5: mild functional impairment, primarily affecting instrumental activities.
• CFS 6 or 7: significant impairment with potential dependency in both instrumental and basic activities.
• CFS 8 or 9: patients dependent in the activities of daily living.

Frailty assessment using the CFS was routinely performed as part of the comprehensive clinical evaluation of patients with ATTR-CA, beginning in September 2019.

### Statistical analysis

Continuous variables are reported as median (Q1-Q3) and were compared between groups using the nonparametric Kruskal-Wallis test, as appropriate. Categorical variables, presented as counts and percentages, were compared using chi-square test or Fisher exact test, as applicable. Ordinal logistic regression was used to identify factors were associated with CFS classes. Results are presented as ORs with 95% CIs. Variables with *P* < 0.10 in the univariable analysis were included in the multivariable logistic regression analysis.

Survival was evaluated using the Kaplan-Meier method and Cox proportional hazards regression analysis, with results reported as HRs with 95% CIs. The proportional hazards assumption was verified using weighted Schoenfeld residuals. Cox multivariable regression analysis was performed to identify factors associated with all-cause mortality, using a backward stepwise elimination method to select variables. Candidate variables with *P* < 0.10 in the univariable analysis were included. Additional models were developed to test the interaction terms, assessing potential effect modification across different baseline characteristics.

The likelihood ratio test was used to evaluate the added prognostic value of incorporating the CFS into the National Amyloidosis Centre (NAC) disease stage model. Harrell’s C-statistic was calculated to measure the discriminatory ability of each model. To compare C-statistics, the dataset was randomly divided into 2 cohorts (1:1); models were fit to the first cohort, and the C-statistics were compared in the second cohort using a 2-sample *t* test with jackknife standard errors.^19^ Sensitivity analyses were performed by censoring patients who were receiving disease-modifying therapies or enrolled in clinical trials at the start date of these interventions. Kaplan-Meier curves were constructed to visualize survival differences between groups, with statistical significance assessed using the log-rank test.

A *P* value of <0.05 was considered statistically significant. All analyses were performed using SPSS Statistics for Macintosh (version 28.0, IBM), STATA (version 18.0, StataCorp), and GraphPad Prism (version 10.1.1, GraphPad Software).

## Results

### Prevalence and distribution of frailty

The study included 880 patients diagnosed with ATTR-CA, comprising 719 (81.7%) with wtATTR-CA, 85 (9.7%) with hATTR-CA due to the p.(V142I) mutation, and 76 (8.6%) with hATTR-CA from non-p.(V142I) mutations. The median age was 80 years (Q1-Q3: 75-84 years), and 763 (86.7%) were male.

The median N-terminal pro–B-type natriuretic peptide (NT-proBNP) level was 2,598 pg/L (Q1-Q3: 1,363-4788 pg/mL), and the median estimated glomerular filtration rate (eGFR) was 59 mL/min/1.73 m^2^ (Q1-Q3: 46-73 mL/min/1.73 m^2^). Most patients were categorized as NAC stage I (51.1%) or NAC stage II (31.9%). During follow-up, 142 (16.1%) patients were enrolled in clinical trials, and 77 (8.8%) initiated disease-modifying therapies, including patisiran (n = 53), tafamidis (n = 14), vutrisiran (n = 9), and inotersen (n = 1). Detailed demographic, clinical, and imaging characteristics are summarized in Table 1. Overall, 43.0% (n = 378) of patients were categorized as CFS 1 to 3. The prevalence of CFS 4 or 5 , CFS 6 or 7, and CFS 8 or 9 was 41.4% (n = 364), 14.7% (n = 129), and 1.0% (n = 9), respectively (Central Illustration, Supplemental Figure 1).

**Table 1:** Clinical and Imaging Characteristics of Patients Diagnosed With ATTR-CM at CFS Assessment

	CFS 1-3 (n = 378, 43.0%)	CFS 4 or 5 (n = 364, 41.4%)	CFS 6 or 7 (n = 129, 14.7%)	CFS 8 or 9 (n = 9, 1.0%)	*P* Value
Demographics					
Age, y	78 (74-82)	82 (77-85)	83 (78-88)	87 (84-90)	<0.001
>80 y	130 (34.3)	200 (55.2)	74 (57.4)	9 (100.0)	<0.001
Men	349 (92.3)	311 (85.2)	95 (73.6)	8 (88.9)	<0.001
Phenotype					
Pure cardiac	356 (94.2)	335 (92.0)	106 (82.2)	9 (100.0)	<0.001
Mixed	22 (5.8)	29 (8.0)	23 (17.8)	0	
ATTR-CA type					
Wild-type	317 (83.9)	303 (83.2)	90 (69.8)	9 (100.0)	<0.001
p.(V142I)	31 (8.2)	42 (11.5)	12 (9.3)	0	
Non-p.(V142I)	30 (7.9)	19 (5.2)	27 (20.9)	0	
Ethnicity^a^					
Caucasian	293/343 (85.4)	272/331 (82.2)	100/115 (86.9)	6/6 (100.0)	0.55
Afro-Caribbean	45/343 (13.1)	53/331 (16.0)	15/115 (13.1)	0	
NYHA functional class III/IV	24 (17.0)	67 (18.4)	45 (34.9)	5 (55.6)	<0.001
NT-proBNP, pg/mL	2,058 (1,065-3,685)	2,919 (1,527-5,976)	3,728 (1,642-6,599)	5,417 (1,065-8,771)	<0.001
NT-proBNP ≥10,000 pg/mL	11 (2.9)	39 (10.7)	17 (13.2)	2 (22.2)	<0.001
eGFR, mL/min/1.73 m^2^	62 (50-77)	58 (45-70)	52 (36-68)	50 (31-64)	<0.001
NAC staging system					
Stage I	236 (62.4)	162 (44.5)	49 (38.0)	3 (33.3)	<0.001
Stage II	101 (26.7)	136 (37.4)	41 (31.8)	3 (33.3)	
Stage III	41 (10.8)	66 (18.1)	39 (30.2)	3 (33.3)	
Ischemic heart disease	67 (17.7)	58 (15.9)	24 (18.6)	1 (11.1)	0.71
Diabetes mellitus	41 (10.8)	83 (22.8)	32 (24.8)	2 (22.2)	<0.001
Hypertension	155 (41.0)	161 (44.2)	55 (42.6)	4 (44.4)	0.54
History of atrial fibrillation	184 (48.7)	181 (49.7)	71 (55.0)	6 (66.7)	0.17
Stroke/TIA	29 (7.7)	39 (10.7)	12 (9.3)	2 (22.2)	0.18
PPM	38 (10.1)	41 (11.3)	22 (17.1)	1 (11.1)	0.11
ICD	9 (2.4)	8 (2.2)	3 (2.3)	0	0.82
Medical therapy					
Beta-blockers	180 (47.6)	171 (47.0)	55 (42.6)	1 (11.1)	0.14
ACE inhibitors/ARBs	159 (42.1)	152 (41.8)	42 (32.6)	0	0.024
MRAs	50 (13.2)	80 (22.0)	22 (17.1)	0	0.15
Loop diuretic	188 (49.7)	214 (58.8)	76 (58.9)	4 (44.4)	0.09
Echocardiographic evaluation^b^					
LV septum, mm	17 (15-19)	17 (15-18)	17 (16-19)	18 (15-19)	0.07
LV posterior wall, mm	16 (14-18)	16 (15-18)	16 (15-18)	17 (15-18)	0.52
LVEF, %	50 (43-58)	49 (40-55)	48 (40-55)	40 (34 or 53)	0.001
E/E′	15.1 (11.7-19.8)	15.7 (12.7-20.3)	16.6 (12.2-21.9)	16.6 (12.1-21.6)	0.21

Values are median (Q1-Q3), n (%), or n/N (%).

ACE = angiotensin converting enzyme; ARB = angiotensin receptor blocker; ATTR-CA = transthyretin cardiac amyloidosis; CFS = Clinical Frailty Scale; E/E′ = E to early diastolic mitral annular tissue velocity; eGFR = estimated glomerular filtration rate; ICD = implantable cardioverter-defibrillator; LV = left ventricular; LVEF = left ventricular ejection fraction; MRA = mineralocorticoid receptor antagonist; NAC = National Amyloidosis Centre; NT-proBNP = N-terminal pro–B-type natriuretic peptide; PPM = permanent pacemaker; TIA = transient ischemic attack.

a Available in 795 patients.

b Available for 861 patients.

**Central Illustration:**
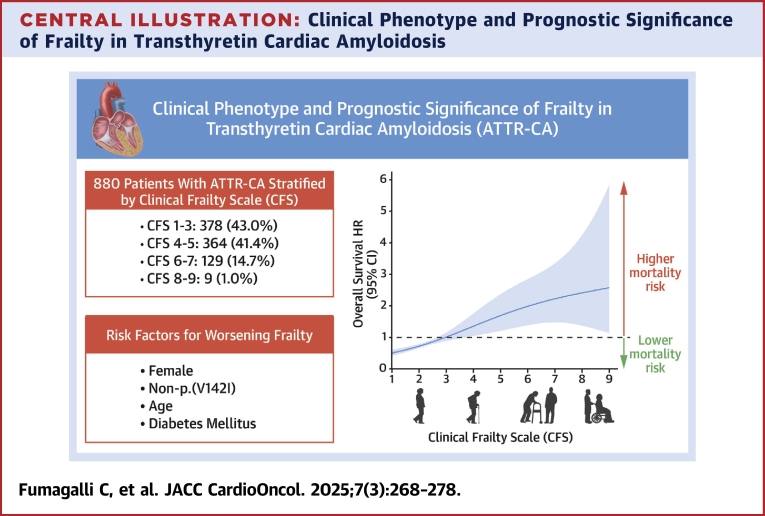
Clinical Phenotype and Prognostic Significance of Frailty in Transthyretin Cardiac Amyloidosis This central illustration depicts the clinical characteristics and prognostic implications of frailty in patients with transthyretin cardiac amyloidosis (ATTR-CA). It includes the distribution of Clinical Frailty Scale (CFS) scores, risk factors associated with worsening frailty, and the relationship between frailty levels and mortality risk. The findings underscore the importance of frailty assessment in risk stratification and optimizing patient management.

**Figure 1:**
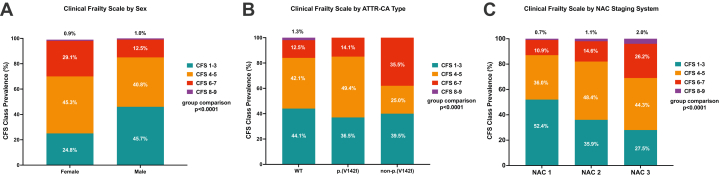
Distribution of CFS Scores Across Different Domains The distribution of patients with transthyretin cardiac amyloidosis (ATTR-CA) across various Clinical Frailty Scale (CFS) categories. (A) Distribution by sex, highlighting any potential sex-related differences in frailty status. (B) Prevalence of CFS groups by ATTR-CA type (wild-type vs hereditary). (C) Distribution according to the National Amyloidosis Centre (NAC) staging system, demonstrating the relationship between disease stage and frailty.

Demographic, clinical, and imaging characteristics stratified by ATTR-CA genotype are provided in Supplemental Tables 2 to 4. The distribution of CFS scores across different domains is presented in Figure 1. Multivariable ordinal logistic regression analysis identified that frailty was independently associated with older age, female sex, non-p.(V142I) hATTR-CA, and progressive NAC stage at baseline (Table 2).

**Table 2:** Baseline Characteristics of ATTR-CA Patients Associated With Clinical Frailty Scale Categories at Ordinal Logistic Regression

	Univariable			Multivariable		
	OR	95% CI	*P* value	OR	95% CI	*P* value
Age (per year increase)	1.071	1.049-1.102	<0.001	1.099	1.082-1.124	<0.001
Male	0.360	0.171-0.589	<0.001	0.301	0.217-0.499	<0.001
Diabetes mellitus	1.899	1.392-2.781	0.010	2.120	1.681-3.001	0.038
Hypertension	1.067	0.838-1.406	0.56			
Atrial fibrillation	1.189	0.914-1.600	0.21			
Ischemic heart disease	0.925	0.691-1.349	0.81			
Mutation status						
p.(V142I) vs wild-type	1.305	0.830-2.190	0.25	0.938	0.609-1.613	0.74
Non-p.(V142I) vs wild-type	1.930	1.018-2.449	<0.001	6.145	3.189-8.795	<0.001
Ejection fraction	0.973	0.929-0.981	0.001	0.971	0.958-0.984	0.007
NAC stage						
II vs I	1.802	1.401-2.592	0.001	1.550	1.189-5.218	0.023
III vs I	3.948	2.051-4.228	<0.001	1.807	1.289-7.375	0.001

Abbreviations as in Table 1.

### Survival

**Figure 2:**
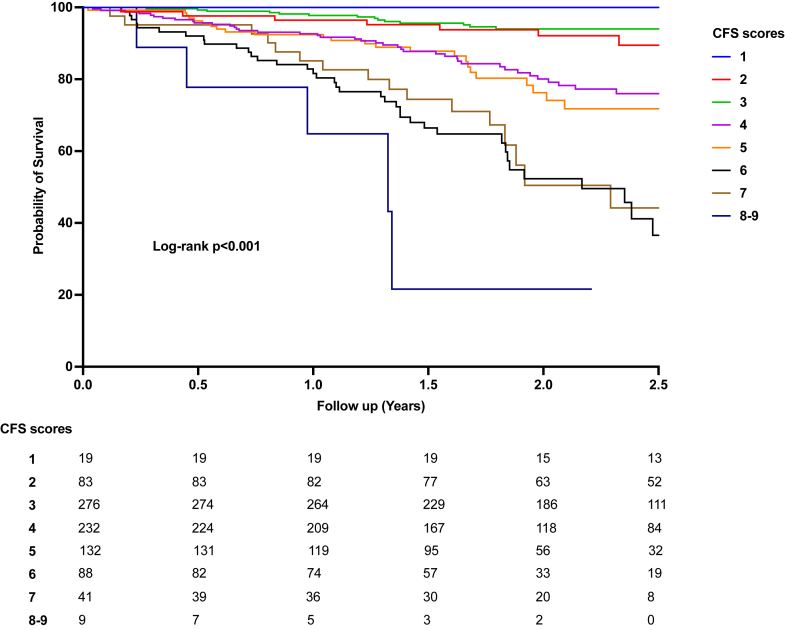
Survival Analysis of Patients With ATTR-CA by Clinical Frailty Status Kaplan-Meier survival curves for patients with ATTR-CA, stratified by frailty status as assessed by the CFS. The figure highlights differences in survival rates using CFS as a continuous variable, emphasizing the prognostic impact of frailty on overall survival. Patients with higher frailty levels have worse survival outcomes, suggesting the importance of frailty assessment in this population. Abbreviations as in Figure 1.

Survival analysis by CFS ordinal classes is presented in Figure 2. The rate of death among patients with a CFS of 1 to 3 was 2.9 per 100 person-years (95% CI: 1.9-4.5 per 100 person-years). In comparison, the death rates were higher for patients with greater frailty: 11.0 per 100 person-years (95% CI: 8.7-14.0 per 100 person-years) for CFS 4 or 5, 21.1 per 100 person-years (95% CI: 16.1-27.7 per 100 person-years) for CFS 6 or 7, and 40.9 per 100 person-years (95% CI: 17.0-98.3 per 100 person-years) for CFS 8 or 9.

In the overall population, frailty was independently associated with higher mortality (Central Illustration). Compared with patients with a CFS of 1 to 3, the HRs were as follows: CFS 4 or 5 (HR: 2.9; 95% CI: 1.7-4.9; *P* < 0.001), CFS 6 or 7 (HR 4.1; 95% CI: 2.4-7.0; *P* < 0.001), and CFS 8 or 9 (HR 9.7; 95% CI: 3.3-28.3; *P* < 0.001). These associations remained significant after adjusting for known predictors, including NAC stage and its components (log-transformed eGFR and NT-proBNP) (Table 3, Supplemental Table 5 for CFS as ordinal, Supplemental Table 6 for WtATTR CA patients only).

**Table 3:** Univariable and Multivariable Cox Regression Analysis to Determine Factors Associated With All-Cause Mortality

	Univariable			Multivariable		
	HR	95% CI	*P* value	HR	95% CI	*P* value
Age at evaluation (Δ year)	1.102	1.071-1.134	<0.001	1.029	0.998-1.061	0.063
Men	1.244	0.728-2.125	0.41			
NYHA functional class (III/IV)	3.162	2.226-4.493	<0.001	1.501	1.038-2.171	0.031
CFS (vs 1-3)			<0.001			<0.001
4 or 5	3.125	2.085-4.971	<0.001	2.941	1.774-4.867	<0.001
6 or 7	4.799	2.666-8.348	<0.001	4.123	2.423-7.015	<0.001
8 or 9	8.038	2.761-19.719	<0.001	9.716	3.340-28.266	<0.001
Log NT-proBNP	2.121	1.428-2.054	<0.001	1.897	1.512-2.379	<0.001
Log eGFR	0.491	0.301-0.894	0.015	0.533	0.311-0.915	0.022
Ischemic heart disease^a^	1.428	0.966-2.110	0.07	1.385	0.932-2.056	0.11
Diabetes mellitus^a^	1.404	0.946-2.082	0.09	1.067	0.719-1.592	0.84
Hypertension^a^	1.061	0.761-1.479	0.73			
Atrial fibrillation	1.330	0.951-1.860	0.09	0.815	0.578-1.152	0.25
Stroke/TIA^a^	1.134	0.653-1.970	0.66			
LV wall thickness (Δ mm)	1.071	1.007-1.138	0.029	1.052	0.966-1.099	0.36
LVEF (Δ %)	0.973	0.958-0.988	0.001	0.998	0.986-1.014	0.81
Loop diuretics^a^	1.381	0.980-1.946	0.07	1.050	0.734-1.509	0.78

The model was performed in 861 individuals.

Abbreviations as in Table 1.

a At the time of assessment.

The likelihood ratio test demonstrated that adding frailty assessment to the NAC disease staging system significantly improved the model’s goodness of fit (chi-square test: 75.01; *P* < 0.001) and discriminatory ability (Harrell’s C-statistic: 0.79 [95% CI: 0.71-0.83] vs 0.69 [95% CI: 0.64-0.75]; *P* < 0.001). Sensitivity analyses, in which patients were censored at the start date of disease-modifying therapy or clinic trial enrollment, confirmed these findings (Supplemental Table 7).

**Figure 3:**
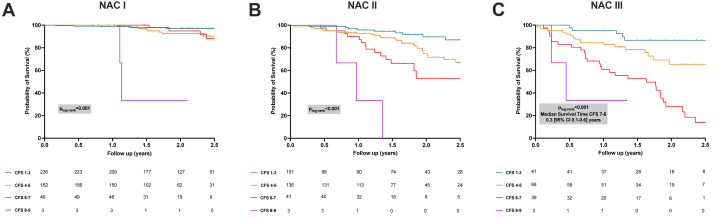
Survival Analysis of Patients With ATTR-CA by NAC Stage and CFS Kaplan-Meier survival curves stratified by the NAC staging system and frailty status. (A) Survival outcomes for patients in NAC stage I across CFS categories. Similar analyses for NAC (B) stage II and (C) stage III. The findings suggest that frailty significantly impacts survival outcomes regardless of disease stage, underscoring its prognostic value. Abbreviations as in Figure 1.

The risk of mortality associated with worsening CFS scores was consistent across all NAC disease stages (Figure 3, Supplemental Figure 2 in patients with a pure cardiac phenotype). In the subgroup of patients with NAC stage III disease (n = 149), those with severe frailty had the poorest prognosis. Median survival was 1.8 years (95% CI: 1.5-2.2 years) for patients with a CFS of 6 or 7 and 0.3 year (95% CI: 0.1-0.6 year) for those with a CFS of 8 or 9. Survival curves in this subgroup diverged significantly at 1 year, with probabilities of survival as follows: 95% (95% CI: 81%-99%) for patients with a CFS of 1 to 3, 83% (95% CI: 71%-90%) for patients with a CFS 4 or 5, 65% (95% CI: 49%-78%) for patients with a CFS 6 or 7, and 18% (95% CI: 1%-51%) for patients with a CFS 8 or 9 (log-rank *P* < 0.001).

**Figure 4:**
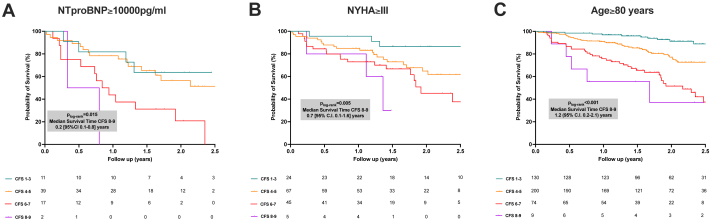
Survival Analysis of Patients With ATTR-CA by NT-proBNP Levels, NYHA Functional Class, and Age Kaplan-Meier survival analysis stratified by N-terminal pro–B-type natriuretic peptide (NT-proBNP) levels, NYHA functional class, and age. (A) Survival differences among patients with NT-proBNP >10,000 pg/mL, indicating worse outcomes at higher levels of frailty. (B) Survival by NYHA functional class, with functional class >III associated with reduced survival. (C) Survival in patients >80 years of age, illustrating the impact of advanced age on prognosis. These analyses highlight the role of clinical factors in determining patient outcomes. Abbreviations as in Figure 1.

Similar results were observed when patients were further stratified by NT-proBNP levels into subgroups of NT-proBNP >10,000 pg/L and NT-proBNP <10,000 pg/L. The increased risk of mortality associated with worsening CFS scores was consistent across both subgroups: NT-proBNP >10,000 pg/L (HR: 2.6; 95% CI: 1.3-5.0; *P* < 0.001) and NT-proBNP <10,000 pg/L (HR: 4.4; 95% CI: 3.0-6.5; *P* < 0.001) (*P* for interaction = 0.58). Notably, among patients with NT-proBNP >10,000 pg/L, those with CFS 8 or 9 had the worst prognosis, with a median survival of 0.2 year (Q1-Q3: 0.1-0.8 year) (Figure 4A, Supplemental Figure 3A).

Survival analysis by CFS in patients with NYHA functional lass ≥III is presented in Figure 4B and Supplemental Figure 3B. The higher risk of mortality associated with worsening CFS scores was consistent in this subgroup, with patients in the CFS 8 or 9 category having the highest risk and a median survival of 0.7 year (95% CI: 0.1-1.6 years). Survival analysis by CFS in patients >80 years of age at the time of evaluation is presented in Figure 4C and Supplemental Figure 3C.

## Discussion

This study comprehensively evaluated the role of frailty in a large cohort of patients with ATTR-CA, demonstrating several key findings. First, the prevalence of frailty was high, with 41.4% of patients categorized as CFS 4 or 5, 14.7% as CFS 6 or 7, and 1.0% as CFS 8 or 9. Second, worsening CFS scores were independently associated with older age, female sex, non-p.(V142I) hATTR-CA variants, and NAC stage III disease. Third, frailty was independently associated with mortality across the entire population and within subgroups defined by age, genotype, disease stage, renal function, NT-proBNP levels, and diuretic use. These findings highlight the importance of frailty assessments for refining risk stratification, with significant implications for clinical practice and the design of clinical trials.

Frailty is a common phenomenon in patients with HF, possibly due to shared pathophysiological pathways, including chronic inflammation, sarcopenia, and reduced physiological reserve.^20^ This study identified older age, female sex, non-p.(V142I) variants, diabetes mellitus, and advanced disease stage as independent predictors of increased CFS scores. These demographic characteristics are consistent with findings in the general HF population, in which older age and female sex are associated with lower skeletal muscle mass, resulting in diminished physiological capacity to handle external stressors.^21^, ^22^, ^23^

In patients with ATTR-CA, the non-p.(V142I) variants were associated with a greater degree of frailty. These differences likely stem from intrinsic variations in disease biology between genotypes, as non-p.(V142I) variants are often associated with concomitant polyneuropathy.^24^^,^^25^ Peripheral sensorimotor neuropathy affects balance, coordination, and mobility, increasing the need for walking aids and assistance with daily activities. Additionally, autonomic neuropathy negatively affects gut motility and can cause malabsorption, leading to malnutrition and sarcopenia, and orthostatic hypotension further exacerbates postural instability, all of which contribute to frailty.^24^^,^^26^ This illustrates the utility of the CFS, which effectively captures the multifaceted and complex nature of the ATTR-CA disease process, including its association with advanced age and multimorbidity, in a single, easily applied, and comprehensive summary measure.

In this large cohort of patients with ATTR-CA, frailty was independently associated with an increased risk of mortality. In cardiovascular medicine, frailty is known to confer a 2-fold increase in mortality, an effect that persists even after adjusting for age and comorbidities.^27^ Recent studies in ATTR-CA have linked frailty to quality of life,^28^ caregiver relationship quality,^29^ and disease duration and severity.^30^ Preliminary evidence also suggests an association between frailty and clinical outcomes.^31^

In our cohort of over 800 patients, frailty had an independent and additive impact on mortality. The CFS demonstrated remarkable capacity to identify patients at the highest risk, with the increased risk associated with severe frailty remaining consistent across genotypes, NAC disease stages, renal function (a key prognostic factor among older individuals), NT-proBNP levels, NYHA functional classes, and age brackets. The CFS is a simple, low-cost, easily performed, and widely applicable tool that complements conventional markers of disease severity, further refining risk stratification.^32^

In conclusion, assessment of frailty, recently linked to health status in ATTR-CA,^28^ may offer valuable prognostic information across genotypes, particularly for estimating short-term risk. In the current era of expanding treatment options for ATTR-CA, incorporating the CFS alongside conventional markers such as NT-proBNP and eGFR could enhance risk stratification, providing incremental information insights beyond genotype and disease severity.^4^^,^^33^

Although the CFS is valuable for identifying patients at higher risk of early mortality, it should not be used in isolation to determine treatment eligibility. Many patients with severe frailty still exhibited outcomes suggesting potential benefit from disease-modifying therapies, underscoring the importance of a comprehensive assessment that integrates frailty with clinical and biomarker data. The primary goal of frailty assessment in ATTR-CA is not to exclude patients from active treatment, but rather to provide a more nuanced understanding of patient risk. This approach can guide discussions on treatment expectations, advanced care planning, and the balance of potential benefits vs risks.^34^ Thus, incorporating the CFS into a multidimensional assessment framework enhances clinical decision making by supporting personalized care while ensuring that access to beneficial therapies is not compromised.^35^

On a broader note, while frailty screening has indeed proven useful for restratifying prognosis, evidence supporting the effectiveness of frailty-based interventions in improving outcomes for elderly patients with cardiovascular disease remains limited. High-quality, large-scale studies are needed to determine the optimal management strategies for these patients and evaluate potential benefits on healthcare-associated costs. Given the high costs of elderly care, interventions aimed at maintaining independence are likely to be more effective.

Future research in ATTR-CA should also investigate the utility and cost-effectiveness of routine vs targeted frailty screening, compare various screening tools, and assess the impact of disease-modifying therapies and frailty-directed interventions on key outcomes such as quality of life, hospitalizations, and survival.

### Study limitations

As a retrospective study, we were able to demonstrate an association between frailty and mortality but could not establish causality. Frailty was assessed with the CFS developed by Rockwood et al,^16^ a tool that evaluates mobility and activities of daily living. However, inter- and intraobserver variability was not assessed.

One of the limitations of the CFS is that it amalgamates several geriatric domains, which may make it less actionable than phenotype-oriented frailty scales that focus on specific attributes such as sarcopenia^36^ or motor capacity (such as the Short Physical Performance Battery). Additionally, other frailty models, such as the Fried frailty phenotype, which incorporates other components like weight loss, exhaustion, low physical activity, slowness, and weakness, were not evaluated in this analysis. While widely used in HF, it remains possible that applying alternative models, such as the Fried physical phenotype,^37^ might have yielded a different prevalence of frailty.

Some patients within the study timeframe were excluded from the analysis due to missing CFS data. Although the number was limited, we cannot rule out the possibility of selection bias. Additionally, information on 6-minute walking distance was available for only a subset of patients and was not included in the final analysis.

Furthermore, female individuals represented <20% of the cohort and were found to be at higher risk of frailty. However, the impact of frailty on outcomes in female is less clear. Finally, this analysis was performed at 2 specialized referral centers for ATTR-CA, and the performance of the CFS in other clinical settings may not be generalizable.

## Conclusions

In this large cohort of patients with ATTR-CA, frailty syndrome (CFS >3) was prevalent and independently associated with an increased risk of mortality. Combining the CFS with conventional markers of disease severity enhanced the precision of risk stratification and identified patients with favorable outcomes, even in advanced disease stages. Future research should focus on a comprehensive assessment of functional, social, and nutritional factors in this population to guide more personalized care strategies.

Perspectives **COMPETENCY IN MEDICAL KNOWLEDGE:** Frailty, a prominent geriatric syndrome, is highly prevalent in patients with ATTR-CA. Although its clinical impact has been poorly described, assessing frailty may improve risk stratification for patients with this condition.

**TRANSLATIONAL OUTLOOK:** Frailty is common in patients with ATTR-CA and independently associated with higher mortality. The CFS effectively restratified outcomes in high-risk categories. Integrating frailty assessment with conventional markers of disease severity can reliably identify high-risk patients with poor prognosis, potentially reducing ageism in care decisions and avoiding therapeutic futility.

## Funding Support and Author Disclosures

Dr Olivotto was supported by grants for “Respect,” “StratifyHF,” and “SmashHCM.” Dr Fontana was supported by a British Heart Foundation Intermediate Clinical Research Fellowship (FS/18/21/33447). Dr Ungar was supported by Piano Nazionale di Ripresa e Resilienza grants. Dr Cappelli has received consulting fees from Pfizer, Alnylam, AstraZeneca, BridgeBio, Daiichi-Sankyo, and Novo Nordisk. Dr Olivotto has received grants from Bayer, MyoKardia (a wholly owned subsidiary of Bristol Myers Squibb), Sanofi Genzyme, and Shire (which is now part of Takeda); personal fees from Bayer, Sanofi Genzyme, and Shire/Takeda; and consulting fees from MyoKardia. Dr Perfetto has received advisory board honoraria from Pfizer, Alnylam, and Akcea. Dr Gillmore has received consulting fees from Alnylam, AstraZeneca, ATTRalus, Bridgebio, Ionis, Intellia, Lycia, and Pfizer. All other authors have reported that they have no relationships relevant to the contents of this paper to disclose.

## References

[1] Ioannou A., Patel R.K., Razvi Y. (2022). Impact of earlier diagnosis in cardiac ATTR amyloidosis over the course of 20 years. Circulation.

[2] Patel R.K., Ioannou A., Razvi Y. (2022). Sex differences among patients with transthyretin amyloid cardiomyopathy – from diagnosis to prognosis. Eur J Heart Fail.

[3] Fumagalli C., Zampieri M., Perfetto F. (2021). Early diagnosis and outcome in patients with wild-type transthyretin cardiac amyloidosis. Mayo Clin Proc.

[4] Gillmore J.D., Judge D.P., Cappelli F. (2024). Efficacy and safety of acoramidis in transthyretin amyloid cardiomyopathy. N Engl J Med.

[5] Garcia-Pavia P., Sultan M.B., Gundapaneni B. (2024). Tafamidis efficacy among octogenarian patients in the phase 3 ATTR-ACT and ongoing long-term extension study. JACC Heart Fail.

[6] Fumagalli C., Maurer M.S., Fontana M. (2024). Comprehensive geriatric assessment to optimize the management of older patients with transthyretin cardiac amyloidosis. JACC Adv.

[7] Hoogendijk E.O., Afilalo J., Ensrud K.E., Kowal P., Onder G., Fried L.P. (2019). Frailty: implications for clinical practice and public health. Lancet.

[8] O’Caoimh R., Sezgin D., O’Donovan M.R. (2021). Prevalence of frailty in 62 countries across the world: a systematic review and meta-analysis of population-level studies. Age Ageing.

[9] Clegg A., Young J., Iliffe S., Rikkert M.O., Rockwood K. (2013). Frailty in elderly people. Lancet.

[10] Ambarish P., Dalane K., Gordon R. (2019). Frailty is intertwined with heart failure. JACC Heart Fail.

[11] Hanlon P., Nicholl B.I., Jani B.D., Lee D., McQueenie R., Mair F.S. (2018). Frailty and pre-frailty in middle-aged and older adults and its association with multimorbidity and mortality: a prospective analysis of 493 737 UK Biobank participants. Lancet Public Health.

[12] Vitale C., Jankowska E., Hill L. (2019). Heart Failure Association of the European Society of Cardiology position paper on frailty in patients with heart failure. Eur J Heart Fail.

[13] Richter D., Guasti L., Walker D. (2022). Frailty in cardiology: definition, assessment and clinical implications for general cardiology. A consensus document of the Council for Cardiology Practice (CCP), Association for Acute Cardio Vascular Care (ACVC), Association of Cardiovascular Nursing and Allied Professions (ACNAP), European Association of Preventive Cardiology (EAPC), European Heart Rhythm Association (EHRA), Council on Valvular Heart Diseases (VHD), Council on Hypertension (CHT), Council of Cardio-Oncology (CCO). Eur J Prev Cardiol.

[14] Garcia-Pavia P., Bengel F., Brito D. (2021). Expert consensus on the monitoring of transthyretin amyloid cardiomyopathy. Eur J Heart Fail.

[15] Gillmore J.D., Damy T., Fontana M. (2018). A new staging system for cardiac transthyretin amyloidosis. Eur Heart J.

[16] Rockwood K. (2005). A global clinical measure of fitness and frailty in elderly people. Can Med Assoc J.

[17] Church S., Rogers E., Rockwood K., Theou O. (2020). A scoping review of the Clinical Frailty Scale. BMC Geriatr.

[18] Rockwood K., Theou O. (2020). Using the Clinical Frailty Scale in allocating scarce health care resources. Can Geriatr J.

[19] Newson R.B. (2010). Comparing the predictive powers of survival models using Harrell’s C or Somers’ D. Stata J.

[20] Beltrami M., Milli M., Fumagalli C. (2021). Frailty, sarcopenia and cachexia in heart failure patients: Different clinical entities of the same painting. World J Cardiol.

[21] Talha K.M., Pandey A., Fudim M., Butler J., Anker S.D., Khan M.S. (2023). Frailty and heart failure: State-of-the-art review. J Cachexia Sarcopenia Muscle.

[22] Forman D.E., Kuchel G.A., Newman J.C. (2023). Impact of geroscience on therapeutic strategies for older adults with cardiovascular disease. J Am Coll Cardiol.

[23] Davis M.R., Lee C.S., Corcoran A., Gupta N., Uchmanowicz I., Denfeld Q.E. (2021). Gender differences in the prevalence of frailty in heart failure: a systematic review and meta-analysis. Int J Cardiol.

[24] Ioannou A., Nitsche C., Porcari A. (2024). Multiorgan dysfunction and associated prognosis in transthyretin cardiac amyloidosis. J Am Heart Assoc.

[25] Lane T., Fontana M., Martinez-Naharro A. (2019). Natural history, quality of life, and outcome in cardiac transthyretin amyloidosis. Circulation.

[26] Porcari A., Razvi Y., Masi A. (2023). Prevalence, characteristics and outcomes of older patients with hereditary versus wild-type transthyretin amyloid cardiomyopathy. Eur J Heart Fail.

[27] Afilalo J., Alexander K.P., Mack M.J. (2014). Frailty assessment in the cardiovascular care of older adults. J Am Coll Cardiol.

[28] Fumagalli C., Ponti L., Smorti M. (2024). Determinants of health status in older patients with transthyretin cardiac amyloidosis: a prospective cohort study. Aging Clin Exp Res.

[29] Fumagalli C., Smorti M., Ponti L. (2023). Frailty and caregiver relationship quality in older patients diagnosed with transthyretin cardiac amyloidosis. Aging Clin Exp Res.

[30] Broussier A., David J.P., Kharoubi M. (2021). Frailty in wild-type transthyretin cardiac amyloidosis: the tip of the iceberg. J Clin Med.

[31] Fine N.M., McMillan J.M. (2021). Prevalence and prognostic significance of frailty among patients with transthyretin amyloidosis cardiomyopathy. Circ Heart Fail.

[32] Denkinger M., Knol W., Cherubini A. (2023). Inclusion of functional measures and frailty in the development and evaluation of medicines for older adults. Lancet Healthy Longev.

[33] Maurer M.S., Schwartz J.H., Gundapaneni B. (2018). Tafamidis treatment for patients with transthyretin amyloid cardiomyopathy. N Engl J Med.

[34] Denfeld Q.E., Jha S.R., Fung E. (2024). Assessing and managing frailty in advanced heart failure: an International Society for Heart and Lung Transplantation consensus statement. The J Heart Lung Transplant.

[35] Krishnaswami A., Steinman M.A., Goyal P. (2019). Deprescribing in older adults with cardiovascular disease. J Am Coll Cardiol.

[36] Afilalo J. (2017). The Clinical Frailty Scale. Circulation.

[37] Fried L.P., Tangen C.M., Walston J. (2001). Frailty in older adults: evidence for a phenotype. J Gerontol A Biol Sci Med Sci.

