# Supplementary material for: Clinical Phenotype and Prognostic Significance of Frailty in Transthyretin Cardiac Amyloidosis
Source: JACC CardioOncol. 2025 Mar 12;7(3):268–78. doi: 10.1016/j.jaccao.2025.01.018 (PMC12046810; doi:10.1016/j.jaccao.2025.01.018)
Supplement: Supplementary Material [file mmc1.docx]

**Clinical Phenotype and Prognostic Significance of Frailty in Transthyretin Cardiac Amyloidosis**

**SUPPLEMENTAL MATERIAL**

| **Supplemental Table 1.**  Clinical Frailty Scale (CFS)  The Clinical Frailty Scale (CFS) is a widely used tool to assess frailty in older adults. It is based on clinical judgment and ranges from 1 (very fit) to 9 (terminally ill). Below is a summary of the categories of the CFS: | | |
| --- | --- | --- |
| Score | Description | Category |
| 1 | Very Fit – People who are robust, active, energetic and motivated. These people commonly exercise regularly. They are among the fittest for their age. | Fit |
| 2 | Well – People who have no active disease symptoms but are less fit than category 1. Often, they exercise or are very active occasionally, e.g., seasonally. | Fit |
| 3 | Managing Well – People whose medical problems are well controlled, but are not regularly active beyond routine walking. | Fit |
| 4 | Vulnerable – While not dependent on others for daily help, often symptoms limit activities. A common complaint is being “slowed up,” and/or being tired during the day. | Mild Frailty |
| 5 | Mildly Frail – These people often have more evident slowing, and need help in high order IADLs (finances, transportation, heavy housework, medica- tions). Typically, mild frailty progressively impairs shopping and walking outside alone, meal preparation and housework. | Mild Frailty |
| 6 | Moderately Frail – People need help with all outside activities and with keeping house. Inside, they often have problems with stairs and need help with bathing and might need minimal assistance (cuing, standby) with dressing. | Severe Frailty |
| 7 | Severely Frail – Completely dependent for personal care, from whatever cause (physical or cognitive). Even so, they seem stable and not at high risk of dying (within ~ 6 months). | Severe Frailty |
| 8 | Very Severely Frail – Completely dependent, approaching the end of life. Typically, they could not recover even from a minor illness. | Severe Frailty |
| 9 | 9.Terminally Ill - Approaching the end of life. This Participating Sites:  category applies to people with a life expectancy <6 months, who are not otherwise evidently frail. | Severe Frailty |

| **Supplemental Table 2. Clinical and imaging characteristics of patients diagnosed with wtATTR-CA at clinical frailty status (CFS) assessment** | | | | | |
| --- | --- | --- | --- | --- | --- |
|  | **CFS 1-3**  **N=317**  **(44.1%)** | **CFS 4-5**  **N=303**  **(42.1%)** | **CFS 6-7**  **N=90**  **(12.5%)** | **CFS 8-9**  **N=9**  **(1.3%)** | **P** |
| **Demographics** |  |  |  |  |  |
| Age, median [Q1-Q3] | 79 [75-82] | 82 [78-85] | 83 [80-88] | 87 [84-90] | <0.001 |
| >80 years, N (%) | 122 (38.4) | 181 (59.7) | 70 (70.7) | 9 (100.0) | <0.001 |
| Men, N (%) | 303 (95.6) | 276 (91.1) | 71 (78.9) | 8 (88.9) | <0.001 |
| Phenotype, N (%) |  |  |  |  |  |
| Pure Cardiac | 317(100.0) | 295 (97.4) | 90 (100.0) | 9 (100.0) | 0.22 |
| Mixed | 0 | 8 (2.6) | 0 | 0 |  |
| NYHA III/IV, N (%) | 20 (6.3) | 59 (19.5) | 37 (41.1) | 5 (55.6) | <0.001 |
| NTproBNP, median [Q1-Q3] | 2138  [1221-3892] | 2941  [1662-5920] | 3648  [1991-7447] | 5417 [1065-8771] | <0.001 |
| NTproBNP >10’000 pg/ml | 9 (2.8) | 32 (10.6) | 11 (12.1) | 2 (22.2) | <0.001 |
| eGFR, median [Q1-Q3] | 63 [51-77] | 57 [45-70] | 45 [34-61] |  | <0.001 |
| NAC Staging System, N (%) |  |  |  |  |  |
| Stage I | 197 (62.1) | 126 (41.6) | 31 (34.4) | 3 (33.3) | <0.001 |
| Stage II | 87 (27.4) | 122 (40.3) | 27 (30.0) | 3 (33.3) |  |
| Stage III | 33 (10.4) | 55 (18.2) | 32 (35.6) | 3 (33.3) |  |
| Ischemic Heart Disease, N (%) | 63 (19.9) | 52 (17.2) | 23 (25.5) | 1 (11.1) | 0.67 |
| Diabetes mellitus, N (%) | 34 (10.7) | 68 (22.4) | 25 (27.7) | 2 (22.2) | <0.001 |
| Hypertension, N (%) | 133 (42.0) | 135 (44.6) | 36 (40.0) | 4 (44.4) | 0.97 |
| History of Atrial Fibrillation, N (%) | 165 (52.1) | 164 (54.1) | 52 (57.7) | 6 (66.7) | 0.27 |
| Stroke/TIA, N (%) | 24 (7.6) | 33 (10.9) | 8 (8.9) | 2 (22.4) | 0.25 |
| PPM, N (%) | 31 (9.8) | 38 (12.5) | 15 (16.7) | 1 (11.1) | 0.07 |
| ICD, N (%) | 7 (2.2) | 7 (2.3) | 2 (2.2) | 0 | 0.96 |
| **Medical Therapy** |  |  |  |  |  |
| Beta-Blockers, N (%) | 157 (49.5) | 143 (47.2) | 41 (45.6) | 1 (11.1) | 0.23 |
| ACEi/ARBs, N (%) | 141 (44.5) | 125 (41.3) | 32 (35.6) | 0 | 0.020 |
| MRAs, N (%) | 43 (13.6) | 66 (21.8) | 18 (20.0) | 0 | 0.026 |
| Loop Diuretic, N (%) | 158 (49.8) | 182 (60.1) | 64 (71.1) | 4 (44.4) | 0.016 |
| **Echocardiographic Evaluation** |  |  |  |  |  |
| LV Septum, median [Q1-Q3], mm | 17 [15-19] | 17 [15-18] | 17 [16-19] | 18 [15-19] | 0.07 |
| LV Posterior Wall, median [Q1-Q3], mm | 16 [14-18] | 16 [15-18] | 16 [15-18] | 17 [15-18] | 0.52 |
| LVEF, median [Q1-Q3], % | 51 [44-58] | 49 [40-55] | 48 [40-56] | 40 [34-53] | 0.001 |
| E/E’, median [Q1-Q3] | 15.0 [11.9-19.2] | 15.7 [12.4-20.3] | 16.6 [12.4-21.6] | 16.6 [12.1-21.6] | 0.21 |
| **ACEi/ARBs:** Angiotensin Converting Enzyme Inhibitor/Angiotensin Receptor Blockers; **ATTR-CA:** Transthyretin Cardiac Amyloidosis; **DMD:** Disease Modifying Drugs; **ICD:** Implantable Cardioverter Defibrillator; **LV:** Left Ventricle; **MRA:** Mineral Corticoid Receptor Antagonist; **NAC:** National Amyloidosis Centre; **NYHA:** New York Heart Association; **PPM:** Permanent Pacemaker; **TIA:** Transient Ischemic Attack; **WT:** wild type; **V:** variant. * Available in 589 patients | | | | | |

| **Supplemental Table 3. Clinical and imaging characteristics of patients diagnosed with p.(V142I) at clinical frailty status (CFS) assessment** | | | | |
| --- | --- | --- | --- | --- |
|  | **CFS 1-3**  **N=31**  **(36.5%)** | **CFS 4-5**  **N=42**  **(49.4%)** | **CFS 6-7**  **N=12**  **(14.1%)** | **P** |
| **Demographics** | | | | |
| Age, median [Q1-Q3] | 75 [71-82] | 80 [77-83] | 82 [75-86] | 0.019 |
| >80 years, N (%) | 8 (25.8) | 17 (40.5) | 7 (58.3) | 0.043 |
| Men, N (%) | 24 (77.4) | 22 (52.4) | 6 (50.0) | 0.034 |
| Phenotype, N (%) |  |  |  |  |
| Pure Cardiac | 29(93.5) | 36 (85.7) | 11 (91.7) | 0.54 |
| Mixed | 2 (6.5) | 6 (14.3) | 1 (8.3) |  |
| NYHA III/IV, N (%) | 2 (6.5) | 7 (16.7) | 3 (25.0) | <0.001 |
| NTproBNP, median [Q1-Q3] | 2580  [859-4525] | 1996  [1110-5242] | 4320  [3521-6266] | 0.19 |
| NTproBNP >10’000 pg/ml | 2 (6.5) | 4 (9.5) | 3 (25.0) | 0.19 |
| eGFR, median [Q1-Q3] | 58 [44-69] | 60 [42-70] | 50 [39-70] | 0.50 |
| NAC Staging System, N (%) |  |  |  |  |
| Stage I | 13 (41.9) | 23 (54.8) | 3 (25.0) | 0.42 |
| Stage II | 11 (35.5) | 9 (21.4) | 4 (33.3) |  |
| Stage III | 7 (22.6) | 10 (23.8) | 5 (41.7) |  |
| Ischemic Heart Disease, N (%) | 4 (12.9) | 6 (14.3) | 1 (8.3) | 0.80 |
| Diabetes mellitus, N (%) | 7 (22.6) | 13 (31.0) | 4 (33.3) | 0.40 |
| Hypertension, N (%) | 19 (61.3) | 22 (52.4) | 8 (66.7) | 0.99 |
| History of Atrial Fibrillation, N (%) | 10 (32.3) | 9 (21.4) | 8 (66.7) | 0.16 |
| Stroke/TIA, N (%) | 3 (9.7) | 4 (9.5) | 3 (25.0) | 0.27 |
| PPM, N (%) | 3 (9.7) | 2 (4.8) | 1 (8.3) | 0.68 |
| ICD, N (%) | 1 (3.2) | 0 (0) | 0 (0) | 0.41 |
| **Medical Therapy** | | | | |
| Beta-Blockers, N (%) | 16 (51.6) | 19 (45.2) | 5 (41.7) | 0.51 |
| ACEi/ARBs, N (%) | 13 (41.9) | 21 (50.0) | 6 (50.0) | 0.54 |
| MRAs, N (%) | 4 (12.9) | 13 (31.0) | 3 (25.0) | 0.19 |
| Loop Diuretic, N (%) | 18 (58.1) | 26 (61.9) | 8 (66.7) | 0.87 |
| **Echocardiographic Evaluation** | | | | |
| LV Septum, median [Q1-Q3], mm | 17 [16-18] | 17 [15-18] | 17 [14-19] | 0.45 |
| LV Posterior Wall, median [Q1-Q3], mm | 17 [15-18] | 16 [15-18] | 17 [14-18] | 0.78 |
| LVEF, median [Q1-Q3], % | 48 [38-51] | 45 [35-53] | 47 [35-51] | 0.92 |
| E/E’, median [Q1-Q3] | 21.0 [12.4-23.6] | 16.6 [14.8-21.5] | 18.5 [12.0-27.1] | 0.97 |
| **ACEi/ARBs:** Angiotensin Converting Enzyme Inhibitor/Angiotensin Receptor Blockers; **ATTR-CA:** Transthyretin Cardiac Amyloidosis; **DMD:** Disease Modifying Drugs; **ICD:** Implantable Cardioverter Defibrillator; **LV:** Left Ventricle; **MRA:** Mineral Corticoid Receptor Antagonist; **NAC:** National Amyloidosis Centre; **NYHA:** New York Heart Association; **PPM:** Permanent Pacemaker; **TIA:** Transient Ischemic Attack; **WT:** wild type; **V:** variant. * Available in 81 patients | | | | |

| **Supplemental Table 4. Clinical and imaging characteristics of patients diagnosed with non-p.(V142I) at clinical frailty status (CFS) assessment** | | | | |
| --- | --- | --- | --- | --- |
|  | **CFS 1-3**  **N=30**  **(39.5%)** | **CFS 4-5**  **N=19**  **(25.0%)** | **CFS 6-7**  **N=27**  **(35.5%)** | **p** |
| **Demographics** | | | | |
| Age, median [Q1-Q3] | 67 [64-72] | 74 [68-77] | 74 [69-79] | 0.003 |
| >80 years, N (%) | 0 | 2 (10.5) | 6 (22.2) | 0.024 |
| Men, N (%) | 22 (73.3) | 13 (68.4) | 18 (66.7) | 0.58 |
| Phenotype, N (%) |  |  |  |  |
| Pure Cardiac | 10 (33.3) | 4 (21.1) | 5 (18.5) | 0.39 |
| Mixed | 20 (66.7) | 15 (78.9) | 22 (81.5) |  |
| NYHA III/IV, N (%) | 2 (6.7) | 1 (5.3) | 5 (18.5) | 0.16 |
| NTproBNP, median [Q1-Q3] | 1341  [859-4525] | 1588  [1110-5242] | 4320  [3521-6266] | 0.18 |
| NTproBNP >10’000 pg/ml | 0 | 3 (15.8) | 3 (11.1) | 0.09 |
| eGFR, median [Q1-Q3] | 58 [44-69] | 60 [42-70] | 50 [39-70] | 0.21 |
| NAC Staging System, N (%) |  |  |  |  |
| Stage I | 26 (86.7) | 13 (68.4) | 15 (55.6) | 0.09 |
| Stage II | 3 (10.0) | 5 (26.3) | 10 (37.0) |  |
| Stage III | 1 (3.3) | 1 (5.3) | 2 (7.4) |  |
| Ischemic Heart Disease, N (%) | 0 | 0 | 0 | - |
| Diabetes mellitus, N (%) | 0 | 2 (10.5) | 3 (11.1) | 0.17 |
| Hypertension, N (%) | 3 (10.0) | 4 (21.1) | 11 (40.7) | 0.023 |
| History of Atrial Fibrillation, N (%) | 9 (30.0) | 8 (42.1) | 11 (40.7) | 0.61 |
| Stroke/TIA, N (%) | 2 (6.7) | 2 (10.5) | 1 (3.7) | 0.66 |
| PPM, N (%) | 4 (13.3) | 1 (5.3) | 6 (22.2) | 0.36 |
| ICD, N (%) | 1 (3.3) | 1 (5.3) | 1 (3.7) | 0.94 |
| **Medical Therapy** | | | | |
| Beta-Blockers, N (%) | 7 (23.3) | 9 (47.4) | 9 (33.3) | 0.22 |
| ACEi/ARBs, N (%) | 5 (16.7) | 6 (31.6) | 4 (14.8) | 0.32 |
| MRAs, N (%) | 3 (10.0) | 1 (5.3) | 1 (3.7) | 0.61 |
| Loop Diuretic, N (%) | 12 (40.0) | 6 (31.6) | 8 (29.6) | 0.69 |
| **Echocardiographic Evaluation** | | | | |
| LV Septum, median [Q1-Q3], mm | 17 [15-18] | 15 [13-17] | 18 [15-19] | 0.040 |
| LV Posterior Wall, median [Q1-Q3], mm | 16 [13-18] | 15 [13-17] | 17 [15-18] | 0.46 |
| LVEF, median [Q1-Q3], % | 55 [50-57] | 55 [47-63] | 50 [39-56] | 0.012 |
| E/E’, median [Q1-Q3] | 16.1 [11.5-18.2] | 15.0 [11.5-18.9] | 14.0 [12.0-20.0] | 0.99 |
| **ACEi/ARBs:** Angiotensin Converting Enzyme Inhibitor/Angiotensin Receptor Blockers; **ATTR-CA:** Transthyretin Cardiac Amyloidosis; **DMD:** Disease Modifying Drugs; **ICD:** Implantable Cardioverter Defibrillator; **LV:** Left Ventricle; **MRA:** Mineral Corticoid Receptor Antagonist; **NAC:** National Amyloidosis Centre; **NYHA:** New York Heart Association; **PPM:** Permanent Pacemaker; **TIA:** Transient Ischemic Attack; **WT:** wild type; **V:** variant. * Available in 56 patients | | | | |

| **Supplemental Table 5. Multivariable Cox regression analysis to determine factors associated with all-cause mortality in patients with ATTR-CA.** | | | |
| --- | --- | --- | --- |
| **Variable** | **HR** | **[95% Conf. Interval]** | **p** |
| **Age** | 1.027 | 0.969-1.059 | 0.08 |
| **NYHA** | 1.501 | 1.037-2.171 | 0.031 |
| **CFS (reference 1)** | |  |  |
| **2** | 2.696 | 0.300-24.215 | 0.38 |
| **3** | 2.048 | 0.271-15.475 | 0.49 |
| **4** | 5.132 | 0.694-37.913 | 0.11 |
| **5** | 7.815 | 1.0453-58.425 | 0.045 |
| **6** | 8.521 | 1.149-63.178 | 0.036 |
| **7** | 8.704 | 1.113-68.020 | 0.039 |
| **8-9** | 20.563 | 2.254-187.563 | 0.007 |
| **Ischemic Heart Disease*** | 1.360 | 0.912-2.021 | 0.13 |
| **Diabetes Mellitus*** | 1.074 | 0.718-1.607 | 0.73 |
| **Atrial Fibrillation** | 0.813 | 0.575-1.149 | 0.24 |
| **LV septal thickness** | 1.030 | 0.964-1.098 | 0.78 |
| **LVEF** | 0.998 | 0.981-1.0138 | 0.79 |
| **Loop diuretics*** | 1.049 | 0.732-1.502 | 0.54 |
| **log NTproBNP** | 1.879 | 1.497-2.356 | <0.001 |
| **log eGFR** | 0.551 | 0.316-0.950 | 0.032 |
| HR: Hazard Ratio; LV: Left Ventricular; NYHA: New York Heart Association Class.  *: At the time of assessment. | | | |

| **Supplemental Table 6. Multivariable Cox regression analysis to determine factors associated with all-cause mortality in patients with wt-ATTR-CA** | | | |
| --- | --- | --- | --- |
|  | **Multivariable** | | |
|  | **HR** | **95% Confidence Interval** | **p** |
| **Age at evaluation (D year)** | 1.038 | 1.001-1.076 | 0.040 |
| **NYHA Class (III/IV)** | 1.378 | 0.920-2.056 | 0.120 |
| **Clinical Frailty Scale (vs 1-3)** |  |  | <0.001 |
| **4-5** | 2.906 | 1.681-5.025 | <0.001 |
| **6-7** | 4.168 | 2.326-7.473 | <0.001 |
| **8-9** | 7.191 | 2.197-23.511 | 0.001 |
| **Log NTproBNP** | 1.871 | 1.454-2.408 | <0.001 |
| **Log eGFR** | 0.506 | 0.287-0.896 | 0.019 |
| **Ischemic Heart Disease*** | 1.378 | 0.923-2.057 | 0.11 |
| **Diabetes mellitus*** | 0.988 | 0.973-1.005 | 0.95 |
| **Atrial Fibrillation** | 0.847 | 0.590-1.216 | 0.37 |
| **LV wall thickness (D mm)** | 1.061 | 0.995-1.131 | 0.07 |
| **LV ejection fraction (D %)** | 0.989 | 0.974-1.005 | 0.14 |
| **Loop Diuretics** | 0.990 | 0.692-1.417 | 0.96 |
| HR: Hazard Ratio; LV: Left Ventricular; NYHA: New York Heart Association Class.  *: At the time of assessment. | | | |

| **Supplemental Table 7. Multivariable Cox regression analysis to determine factors associated with all-cause mortality in patients with transthyretin cardiac amyloidosis (ATTR-CA) censored at** **the start date of disease modifying therapy or clinic trials.** | | | |
| --- | --- | --- | --- |
|  | **Multivariable** | | |
|  | **HR** | **95% Confidence Interval** | **p** |
| **Age at evaluation (D year)** | 1.039 | 1.003-1.076 | 0.033 |
| **NYHA Class (III/IV)** | 1.359 | 1.109-2.911 | 0.018 |
| **Clinical Frailty Scale (vs 1-3)** |  |  | <0.001 |
| **4-5** | 2.926 | 1.698-5.055 | <0.001 |
| **6-7** | 4.120 | 2.301-7.377 | <0.001 |
| **8-9** | 6.954 | 2.125-22.769 | <0.001 |
| **Log NTproBNP** | 1.821 | 1.418-2.342 | <0.001 |
| **Log eGFR** | 0.509 | 0.286-0.897 | 0.016 |
| **Ischemic Heart Disease*** | 1.148 | 0.773-1.794 | 0.54 |
| **Diabetes mellitus*** | 0.812 | 0.614-1.465 | 0.81 |
| **Atrial Fibrillation** | 0.729 | 0.401-1.398 | 0.77 |
| **LV wall thickness (D mm)** | 1.021 | 0.919-1.134 | 0.31 |
| **LV ejection fraction (D %)** | 0.988 | 0.971-1.006 | 0.20 |
| **Loop Diuretics*** | 1.095 | 0.736-1.627 | 0.65 |
| HR: Hazard Ratio; LV: Left Ventricular; LV: Left Ventricle; NYHA: New York Heart Association.  *: At the time of assessment. | | | |

| **Supplemental Table 8. Multivariable Cox regression analysis to determine factors associated with all-cause mortality in patients with transthyretin cardiac amyloidosis (ATTR-CA) with adjustment for the NAC Staging system.** | | | |
| --- | --- | --- | --- |
|  | **Multivariable** | | |
|  | **HR** | **95% Confidence Interval** | **p** |
| **Age at evaluation (D year)** | 1.031 | 1.002-1.060 | 0.034 |
| **NYHA Class (III/IV)** | 1.528 | 1.056-2.201 | 0.024 |
| **Clinical Frailty Scale (vs 1-3)** |  |  | <0.001 |
| **4-5** | 2.693 | 1.438-5.612 | <0.001 |
| **6-7** | 4.343 | 2.000-7.571 | <0.001 |
| **8-9** | 7.331 | 2.912-24.819 | <0.001 |
| **NAC II vs I** | 2.241 | 1.433-3.503 | <0.001 |
| **NAC III vs I** | 4.218 | 2.656-6.698 | 0.001 |
| **Ischemic Heart Disease*** | 1.292 | 0.871-1.916 | 0.88 |
| **Diabetes mellitus*** | 0.971 | 0.648-1.453 | 0.34 |
| **Atrial Fibrillation** | 0.855 | 0.976-1.201 | 0.19 |
| **LV wall thickness (D mm)** | 1.044 | 0.979-1.111 | 0.29 |
| **LV ejection fraction (D %)** | 0.991 | 0976-1.008 | 0.31 |
| **Loop Diuretics*** | 1.031 | 0.724-1.466 | 0.86 |
| HR: Hazard Ratio; LV: Left Ventricle; NAC: National Amyloidosis Centre; NYHA: New York Heart Association.  *: At the time of assessment. | | | |

| Supplemental Figure 1 |
| --- |
| 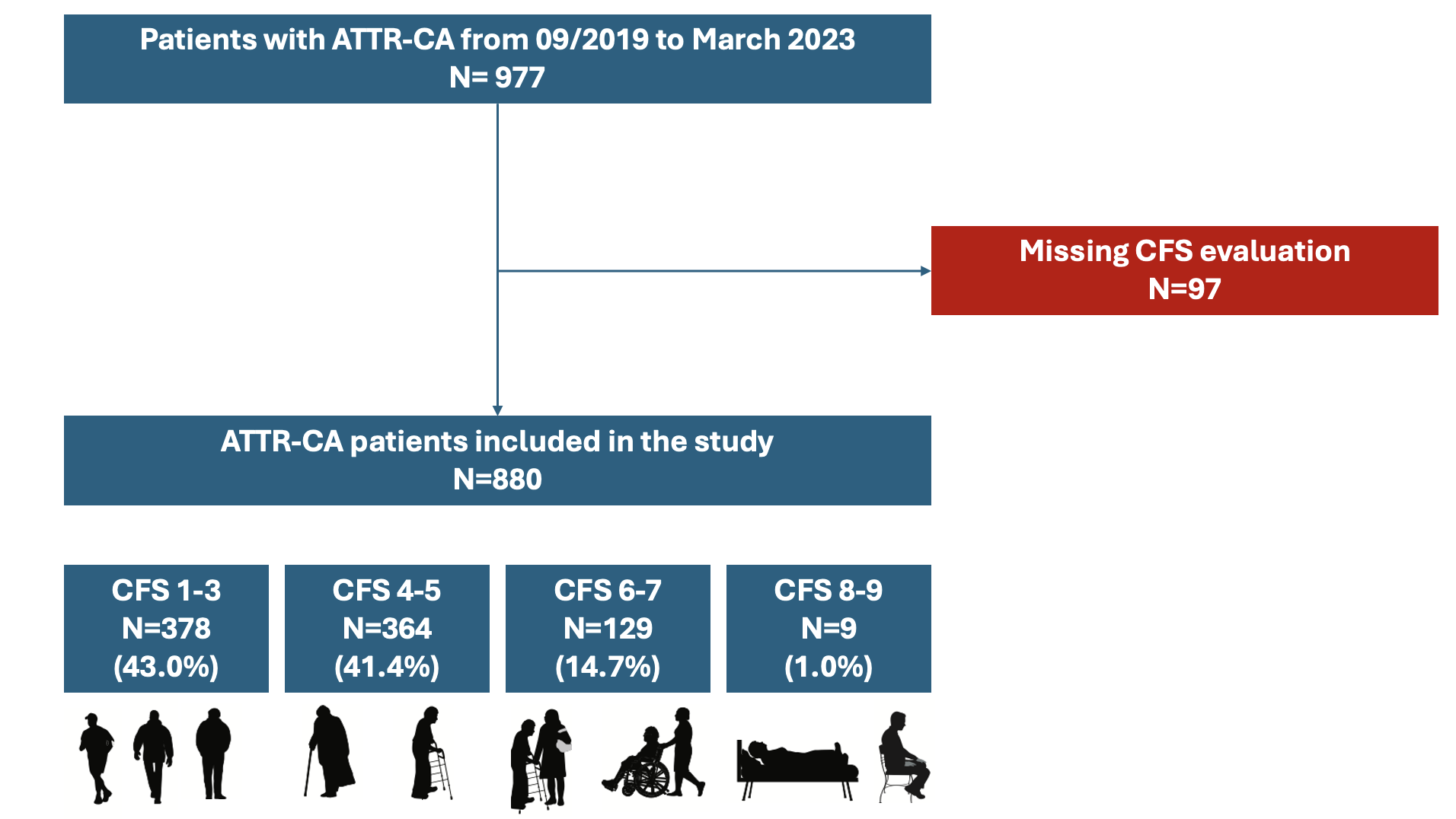 |
| **Supplemental Figure 1. Study Population Flowchart.**  **Caption: CONSORT diagram of patients diagnosed with ATTR-CA and screened for presence of CFS. ATTR-CA= Transthyretin Cardiac Amyloidosis; CFS=Clinical Frailty Scale; NAC: National Amyloidosis Centre.**  **Supplemental Figure 2** |
|  |
|  |
|  |

**Supplemental Figure 2**. Survival Analysis of patients with ATTR-CA by NAC Score and CFS in patients with a pure cardiac phenotype. Panel A: Patients in NAC Stage I; Panel B: Patients in NAC Stage II; Panel C: Patients in NAC Stage III. Legend: ATTR-CA= Transthyretin Cardiac Amyloidosis; CFS=Clinical Frailty Scale; NAC: National Amyloidosis Centre.

| **Supplemental Figure 3** |
| --- |
|  |

**Supplemental Figure 3**. Survival Analysis of patients with ATTR-CA and a pure cardiac phenotype. Panel A: Patients with NTproBNP>10’000 pg/ml; Panel B: Patients in NYHA Class>III; Panel C: Patients with Age>80 years
